# Effects of Rosmarinic Acid on L‐Methionine‐Induced Behavioral Impairments and Disruptions in Hippocampal Neurogenesis in the Adult Rat

**DOI:** 10.1002/fsn3.72502

**Published:** 2026-10-26

**Authors:** Salinee Naewla, Jirawadee Chaithum, Tanaporn Anosri, Nataya Sritawan, Anusara Aranarochana, Jariya Umka Welbat, Peter Wigmore, Apiwat Sirichoat

**Affiliations:** ^1^ Department of Basic Medical Science, Faculty of Medicine Vajira Hospital Navamindradhiraj University Bangkok Thailand; ^2^ Neurogenesis Research Group, Department of Anatomy, Faculty of Medicine Khon Kaen University Khon Kaen Thailand; ^3^ Department of Anatomy, Faculty of Medicine Khon Kaen University Khon Kaen Thailand; ^4^ Faculty of Medicine Mahasarakham University Mahasarakham Thailand; ^5^ School of Life Sciences, Medical School, Queen's Medical Centre University of Nottingham Nottingham UK

**Keywords:** hippocampus, L‐methionine, memory, neurogenesis, Rosmarinic acid

## Abstract

L‐methionine (L‐met) is an essential amino acid; however, excessive intake induces oxidative stress associated with memory impairment and reduced hippocampal neurogenesis. Rosmarinic acid (RA), a natural phenolic compound, has been reported to exhibit antioxidant and neuroprotective properties. This study aimed to investigate the effects of RA on L‐met‐induced memory deficits and alterations in hippocampal neurogenesis in adult rats. Fifty‐four male Sprague–Dawley rats were divided into 6 groups: control, L‐met (1.7 g/kg/day), RA10 (10 mg/kg/day), RA30 (30 mg/kg/day), L‐met + RA10 (L‐met: 1.7 g/kg/day and RA: 10 mg/kg/day), and L‐met + RA30 (L‐met: 1.7 g/kg/day and RA: 30 mg/kg/day). Memory performance was evaluated using the novel object location (NOL) and novel object recognition (NOR) tests. Hippocampal neurogenesis was assessed using Ki‐67 and Ki‐67/rat endothelial cell antigen‐1 (RECA‐1) as markers of cell proliferation and vascular‐associated proliferating cells, 5‐bromo‐2′‐deoxyuridine/neuronal nuclear protein (BrdU/NeuN) as a marker of neuronal survival associated with mature neurons and p21 as a marker of cell cycle arrest. The results revealed that L‐met administration significantly impaired memory performance, reduced Ki‐67‐, Ki‐67/RECA‐1‐, and BrdU/NeuN‐positive cells, and increased p21‐positive cells. However, co‐administration of RA (10 and 30 mg/kg) significantly attenuated L‐met‐induced memory deficits and hippocampal neurogenesis‐related impairments. These findings suggest that RA attenuates L‐met‐induced memory impairment and disruption of hippocampal neurogenesis in adult rats.

## Graphical abstract

“Excessive L‐methionine (L‐met) intake impairs spatial (NOL) and recognition (NOR) memory, suppresses cell proliferation and vascular‐associated proliferating cells (Ki‐67 and Ki‐67/RECA‐1) and neuronal survival (BrdU/NeuN), and increases cell‐cycle arrest (p21) in the hippocampal dentate gyrus of adult rats. Co‐administration of Rosmarinic acid (RA) attenuates L‐met‐induced neurotoxicity, restoring cognitive performance and maintaining adult hippocampal neurogenesis.”

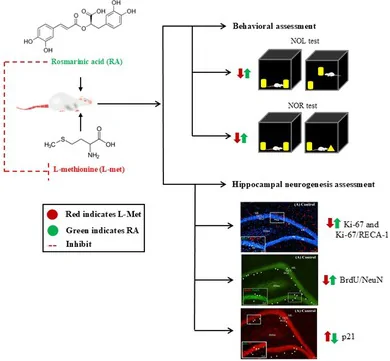

## 1 Introduction

The World Health Organization predicts that 78 million people will be living with dementia worldwide by 2030, increasing to nearly 139 million by 2050 (Shin 2022). Dementia significantly affects quality of life, including cognitive function, occupational performance, and participation, and also increases the burden on caregivers (Millán‐Calenti et al. 2009; Park et al. 2013; Parle and Singh 2007). Dementia is closely linked to Alzheimer's disease (AD), which is characterized by behavioral alterations and memory loss (Cam and Bu 2006). Studies have shown that memory impairment and cognitive decline are closely associated with reduced neurogenesis (Hernández‐Mercado and Zepeda 2021).

Adult hippocampal neurogenesis is a complex process involving the generation of new neurons from neural stem cells (NSCs) and neural progenitor cells (NPCs) in the dentate gyrus (DG) of the hippocampus. This process consists of several stages, including proliferation, differentiation, migration and maturation (Eriksson et al. 1998). Therefore, enhancing neurogenesis is essential for maintaining cognitive function and is considered a potential therapeutic strategy for dementia. Importantly, both intrinsic and extrinsic factors influence adult neurogenesis, including neurotrophic factors, inflammatory cytokines, physical activity, and dietary intake (D. N. Abrous et al. 2005; Kohman and Rhodes 2013; Lee et al. 2002). Among these, dietary factors have gained increasing attention due to their potential health benefits and modulatory effects on brain function.

L‐methionine (L‐met) is an essential amino acid that plays a crucial role in various metabolic pathways (Orgeron et al. 2014). It is primarily found in animal‐based foods, such as meat, fish, and milk, as well as certain plant‐based sources, including nuts and seeds (Kalhan and Marczewski 2012). However, excessive intake of L‐met has been associated with neurotoxic effects (Ingenbleek and Kimura 2013). L‐met is metabolized into S‐adenosylmethionine (SAM) and subsequently converted into homocysteine (Hcy) (Bottiglieri 2002). Homocysteine, a non‐essential amino acid, is a byproduct of L‐met metabolism. Notably, long‐term consumption of high levels of L‐met can lead to hyperhomocysteinemia (HHcy), a condition associated with cerebrovascular disease, brain aging, and vascular dementia. Previous studies have reported that HHcy can induce oxidative stress, mitochondrial dysfunction, and inflammation, all of which contribute to impaired cognitive function and reduced neurogenesis (Obeid and Herrmann 2006; Sudduth et al. 2013). Therefore, understanding how to counteract L‐met induced neurotoxicity is crucial for developing potential interventions to prevent the deterioration of neuronal function.

Rosmarinic acid (RA, C_18_H_16_O_8_) is a phenolic compound that is widely found in plants, particularly in the *Lamiaceae* family, including rosemary, *Ocimum tenuiflorum* , *Perilla frutescens* , *Salvia miltiorrhiza* Bunge, and lemon balm (Amoah et al. 2016). RA is known for its diverse biological activities, including anti‐inflammatory, antioxidant and neuroprotective effects (Adomako‐Bonsu et al. 2017; J. Wang et al. 2012). Several studies have highlighted the beneficial effects of RA in enhancing memory and alleviating neurotoxicity (Fonteles et al. 2016; Hasanein et al. 2017). Additionally, RA has been shown to suppress amyloid‐β (Aβ) aggregation, a key pathological feature of AD (Hase et al. 2019; Yamamoto et al. 2021). RA also modulates γ‐aminobutyric acid (GABA) receptor activity, which may contribute to its effects on neuronal function and learning, memory (Wang, Hsieh, et al. 2021). These findings suggest that RA may have beneficial effects on hippocampal neurogenesis and cognitive function.

Despite these findings, the effects of RA against L‐met induced impairments, particularly in memory performance and hippocampal neurogenesis, remain unclear. Moreover, its potential role in vascular‐associated cell proliferation and cell cycle–related processes remains unclear. Therefore, the present study aimed to investigate the effects of RA on L‐met induced memory deficits and hippocampal neurogenesis disruption in a rat model. This study assessed memory‐related behavioral performance and investigated hippocampal neurogenesis using microscopic analysis. Behavioral performance was evaluated using the novel object location (NOL) and novel object recognition (NOR) tests, while neurogenesis was assessed through markers of cell proliferation (Ki‐67 and Ki‐67/rat endothelial cell antigen‐1; Ki67/RECA‐1), neuronal survival (5‐bromo‐2′‐deoxyuridine/neuronal nuclear protein; BrdU/NeuN), and cell cycle arrest (p21). The findings of this study are expected to provide further evidence supporting the cognitive and neurogenic benefits of RA.

## 2 Materials and Methods

### 2.1 Animals

Male Sprague–Dawley rats, aged 4–6 weeks (180–200 g), were obtained from Nomura Siam International Co. Ltd. in Bangkok, Thailand. The rats were maintained under standard laboratory conditions at 23°C ± 2°C with a 12‐h light/dark cycle and unrestricted access to standard pellet feed and water. Ethical approval for the study was granted by the Animal Ethics Committee of Khon Kaen University (AICUC‐KKU‐70/2565).

### 2.2 Drugs and Treatment Protocols

L‐met (Sigma‐Aldrich, Merck, Darmstadt, Germany) was prepared as a suspension in 0.1% w/v carboxymethyl cellulose (CMC). RA (Sigma‐Aldrich, Merck, Darmstadt, Germany) was dissolved in propylene glycol and administered orally by gavage at doses of 10 and 30 mg/kg body weight. A total of 54 rats were randomly allocated into six experimental groups. The control group received propylene glycol (1 mL/kg/day) and CMC (5 mL/kg/day). The L‐met group received L‐met (1.7 g/kg/day). The RA10 group received RA (10 mg/kg/day). The RA30 group received RA (30 mg/kg/day). The L‐met + RA10 group received L‐met (1.7 g/kg/day) and RA (10 mg/kg/day), while the L‐met + RA30 group received L‐met (1.7 g/kg/day) and RA (30 mg/kg/day), all administered orally. All treatments were administered orally by gavage once daily for 28 consecutive days. The timeline for all experimental procedures, treatment protocols, and behavioral tests is illustrated in Figure 1.

**FIGURE 1:**
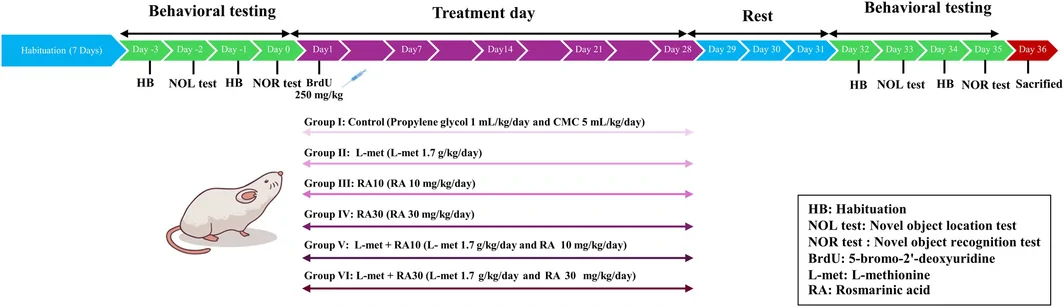
Timeline of the experimental protocol.

Chronic L‐met administration has previously been used as an experimental model associated with HHcy, cognitive impairment, and hippocampal alterations in rodents (Hemanth Kumar et al. 2017; Kovalska et al. 2021; Obeid and Herrmann 2006; Sudduth et al. 2013).

In addition, BrdU (Sigma‐Aldrich, St. Louis, MO, USA) was administered as a single intraperitoneal (i.p.) injection on the first day of the experiment at a dose of 250 mg/kg body weight.

### 2.3 Behavioral Testing

The NOL and NOR tests were selected to assess spatial and recognition memory (Dix and Aggleton 1999; Naewla et al. 2019; Umka et al. 2010). After the 28‐day treatment period, rats were subjected to two behavioral tasks: the NOL test to evaluate spatial memory and the NOR test to assess recognition memory. Behavioral performance was evaluated in an open‐field arena (50 × 50 × 50 cm; black plastic box) containing two objects. A video camera positioned above the arena was connected to the EthoVision XT tracking software (version 12; Noldus, Wageningen, Netherlands). The objects and arena were wiped with 20% ethanol between trials to eliminate any olfactory cues.

#### 2.3.1 NOL Test

Rats were habituated to the empty arena for 30 min. On the following day, each rat was placed in the open‐field box and allowed to freely investigate two identical objects positioned at locations A and B for 3 min during the familiarization trial. Rats were then returned to their home cages for a 15‐min retention period. During the choice trial, rats were reintroduced to the arena, in which one object remained in its familiar location (FL), while the other was moved to a novel location (NL). Each rat was permitted 3 min to explore the two objects.

#### 2.3.2 NOR Test

The NOR test followed a similar procedure. On the following day, rats were introduced into the arena and allowed to interact with the two identical objects (A and B) for 3 min during the familiarization trial. Rats were then returned to their home cages for a 15‐min retention period. During the choice trial, rats were reintroduced to the arena, where one familiar object (FO, object A) and one novel object (NO, object C) were positioned in the same locations as in the prior familiarization trial. Each rat was permitted 3 min to explore the arena with the 2 objects.

Exploration was defined as the rat's nose approaching within 2 cm of an object, involving sniffing or touching. The preference index (PI) was calculated as follows: PI = (time spent exploring the NL or NO/total exploration time) × 100. The data were expressed as percentages and compared with the 50% chance level.

Locomotor activity, including distance moved and velocity, was recorded during the behavioral testing procedures to determine whether differences in general motor activity could confound the interpretation of cognitive performance.

### 2.4 Brain Tissue Preparation

Following behavioral testing, rats were deeply anesthetized to minimize pain and distress and then euthanized by rapid stunning followed by cervical dislocation. After dissection, the brains were quickly isolated and bisected into two hemispheres before cryoprotection in 30% sucrose dissolved in phosphate‐buffered saline (PBS). The hemispheres were then embedded in optimal cutting temperature (OCT) compound (Sakura Finetek, Torrance, CA, USA), rapidly frozen in isopentane cooled with liquid nitrogen and stored at −80°C for immunofluorescence studies. Coronal sections of the DG were cut at 20 μm and 40 μm thick sections using a cryostat (Microm International GmbH, Walldorf, Germany).

### 2.5 Immunofluorescence Staining

Cell proliferation and vascular‐associated proliferating cells were identified using Ki‐67 and Ki‐67/RECA‐1 staining. The right hemisphere was serially sectioned into coronal slices (20‐μm‐thick) spanning from Bregma −2.3 to −6.3 mm to encompass the DG. A total of nine coronal sections per brain were collected by selecting every 15th section throughout the entire DG. Briefly, the sections were fixed in 0.5% paraformaldehyde (PFA; Sigma‐Aldrich Merck, Darmstadt, Germany) for 3 min, followed by incubation with an anti‐Ki‐67 primary antibody (1:150, NCL‐L‐Ki67‐MM1, Novocastra, Leica Biosystems, Newcastle, UK) for 1 h. Sections were washed with PBS and incubated with rabbit anti‐mouse IgG secondary antibody (1:250, A11059, Invitrogen, Thermo Fisher Scientific, Waltham, MA, USA) for 1 h. After washing, sections were incubated with anti‐RECA‐1 primary antibody (1:100, sc‐52,665, Santa Cruz Biotechnology, Dallas, TX, USA) for 1 h. Sections were then incubated with goat anti‐rat IgG secondary antibody (1:250, A11077, Invitrogen, Thermo Fisher Scientific, Waltham, MA, USA) for 1 h. Sections were washed three times with PBS, counterstained with DAPI (1:6000, Molecular Probes, Thermo Fisher Scientific, Waltham, MA, USA) and mounted on glass slides.

Survival of newly generated neuronal cells was examined by BrdU/NeuN staining. The left hemisphere was serially sectioned into coronal slices (each 40 μm‐thick) spanning from Bregma −2.3 to −6.3 mm. A total of nine coronal sections per brain were collected by selecting every 8th section throughout the entire DG. Briefly, sections were incubated overnight at 4°C with anti‐BrdU primary antibody (1:400, ab8152, Abcam, Cambridge, UK). After washing with PBS, sections were incubated with goat anti‐rabbit IgG secondary antibody (1:400, A11011, Invitrogen, Thermo Fisher Scientific, Waltham, MA, USA) for 2 h. Sections were then incubated with anti‐NeuN primary antibody (1:500, MAB377, Merck, Darmstadt, Germany) at 4°C overnight. The following day, sections were incubated with rabbit anti‐mouse IgG secondary antibody (1:500, A11059, Invitrogen, Thermo Fisher Scientific, Waltham, MA, USA) for 2 h. And then mounted using glycerol.

Cell cycle arrest was examined by p21 staining. Coronal sections (40 μm‐thick) were incubated overnight at 4°C with p21 primary antibody (1:100; sc‐397, Santa Cruz Biotechnology, Dallas, TX, USA). After washing with PBS, sections were incubated with a goat anti‐mouse secondary antibody (1:300, A11059, Invitrogen, Thermo Fisher Scientific, Waltham, MA, USA) for 1 h. Sections were counterstained with PI and mounted on glass slides.

### 2.6 Microscopic Quantification and Cell Counting

Quantification of Ki‐67‐, Ki‐67/RECA‐1‐, BrdU/NeuN‐ and p21‐positive cells in the DG was performed on nine sections per rat. Images were captured and analyzed using a fluorescence microscope (Nikon Eclipse 80i, Melville, NY, USA) with 20X and 40X objective lenses. Fluorescein isothiocyanate (FITC) filter was used to detect Ki‐67‐, NeuN‐ and p21‐positive cells. Tetramethylrhodamine isothiocyanate (TRITC) filter was used to detect BrdU‐, RECA‐1‐positive cells, as well as nuclei stained with propidium iodide. DAPI filter was used to detect nuclear staining.

Ki‐67‐ and Ki‐67/RECA‐1‐positive cells were quantified within 3 cell body diameters from the inner boundary of the DG, corresponding to the subgranular zone (SGZ). Ki‐67‐positive cells located within approximately two cell body diameters of RECA‐1‐positive cells were classified as vascular‐associated, whereas the remaining cells were classified as non‐vascular‐associated. Total Ki‐67 counts were obtained by summing both vascular‐associated and non‐vascular‐associated counts across nine sections and were subsequently multiplied by 15 to estimate cell proliferation. p21‐positive cells were counted within the SGZ, defined as three cell body diameters from the inner edge of the DG. BrdU/NeuN‐positive cells were quantified within the SGZ and granule cell layer. The number of BrdU/NeuN‐ and p21‐positive cell counts from every 8th section were summed and multiplied by 8, as previously described (Sirichoat et al. 2024).

### 2.7 Statistical Analysis

Behavioral testing and immunofluorescence outcomes were analyzed using GraphPad Prism (version 11.0; GraphPad Software Inc., San Diego, CA, USA) and reported as mean ± standard error of the mean (SEM). Within‐group differences in object exploration time between the familiarization and choice sessions were analyzed using a paired Student's *t*‐test. Preference index (PI) values were compared with the chance level (50%) using a one‐sample *t*‐test. All other data were analyzed using one‐way analysis of variance (ANOVA), followed by Bonferroni post hoc test. A value of *p* < 0.05 was considered statistically significant.

## 3 Results

### 3.1 Effects of RA and L‐Met on Behavior

#### 3.1.1 NOL Test

Locomotor activity was assessed by distance moved and velocity. There were no significant differences among groups (distance moved: *F* (5, 30) = 1.184, *p* = 0.3405; velocity: *F* (5, 30) = 2.045, *p* = 0.1006, one‐way ANOVA; Figure 2, Table 1), indicating that RA and L‐met did not affect locomotor activity. During the familiarization trial, no significant differences were observed among the groups in the time spent exploring the identical objects placed in different locations (*p* > 0.05, paired Student's *t*‐test, Figure 2), suggesting no inherent location preference. In the choice trial, rats in the control, RA (10 and 30 mg/kg) and L‐met + RA groups showed a significant preference for the object in the novel location compared to the familiar location (Control: *p* < 0.05; RA10: *p* < 0.05; RA30: *p* < 0.01; L‐met + RA10: *p* < 0.05, and L‐met + RA30: *p* < 0.05, paired Student's *t*‐test, Figure 2). In contrast, the L‐met group failed to discriminate between locations (*p* > 0.05), indicating impaired spatial memory. Consistently, the PI in control, RA (10 and 30 mg/kg) and L‐met + RA (10 and 30 mg/kg) groups was significantly higher than the 50% chance level (Control: *p* < 0.05; RA10: *p* < 0.05; RA30: *p* < 0.01; L‐met + RA10: *p* < 0.01, and L‐met + RA30: *p* < 0.01, one‐sample *t*‐test, Figure 2), whereas the L‐met group did not differ from chance level (*p* > 0.05). These results indicate that L‐met induces spatial memory impairment, which is attenuated by RA treatment.

**FIGURE 2:**
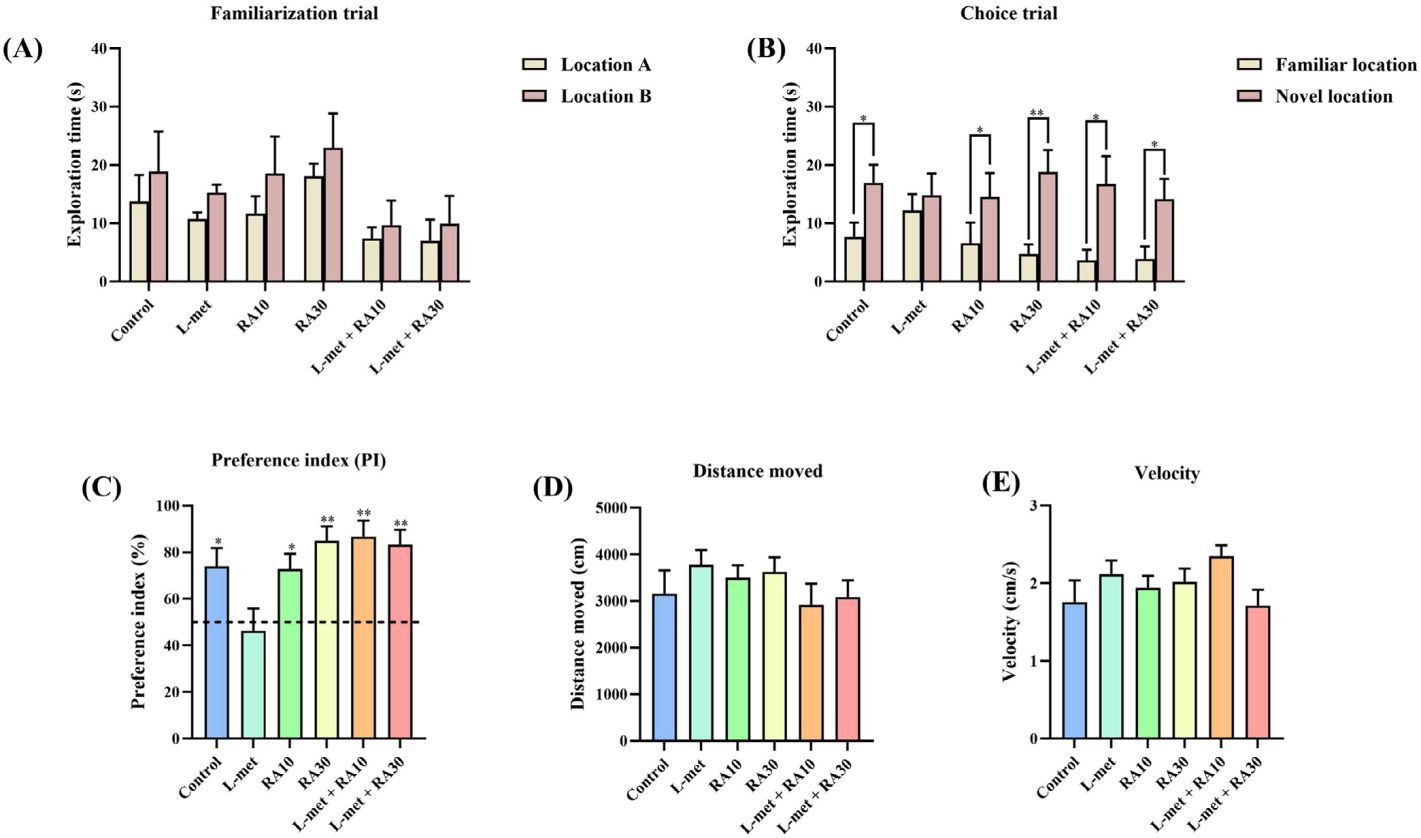
Exploration time during the familiarization (A) and choice trials (B) in the NOL test. No significant differences were observed during familiarization (*p* > 0.05). In the choice trial, all groups except the L‐met group showed a preference for the novel location (*p < 0.05 and **p < 0.01). The PI (C) was significantly above the 50% chance level in all groups except the L‐met group (*p < 0.05 and **p < 0.01). Distance moved (D) and velocity (E) did not differ significantly among the groups (*p* > 0.05). Data are presented as mean ± SEM.

**TABLE 1:** Distance moved and velocity during the habituation phase of the NOL and NOR tests following RA and L‐met administration.

Group	NOL test		NOR test	
	Distance moved (cm)	Velocity (cm/s)	Distance moved (cm)	Velocity (cm/s)
Control	2589 ± 580.0	1.837 ± 0.236	3311 ± 458.3	1.545 ± 0.1066
L‐met	3786 ± 424.9	2.106 ± 0.236	3598 ± 415.9	2.023 ± 0.244
RA10	3738 ± 294.1	2.079 ± 0.163	3598 ± 392.1	1.916 ± 0.1801
RA30	3378 ± 354.2	1.878 ± 0.196	3149 ± 278.2	1.544 ± 0.110
L‐met + RA10	3019 ± 553.4	2.441 ± 0.170	3468 ± 304.2	1.515 ± 0.154
L‐met + RA30	2851 ± 412.9	1.585 ± 0.2295	2799 ± 498.9	1.588 ± 0.2692

*Note:* Data are represented as mean ± SEM.

#### 3.1.2 NOR Test

No significant differences in distance moved and velocity were observed among groups (distance moved: *F* (5, 30) = 0.5985, *p* = 0.7014; velocity: *F* (5, 30) = 1.387, *p* = 0.2572, one‐way ANOVA; Figure 3, Table 1), suggesting that neither RA nor L‐met affected locomotor activity. During the familiarization trial, all groups displayed comparable exploration of identical objects (*p* > 0.05, paired Student's *t*‐test; Figure 3). In the choice trial, rats in the control, RA (10 and 30 mg/kg), and L‐met + RA (10 and 30 mg/kg) groups spent significantly more time exploring the novel object than the familiar one (Control: *p* < 0.01; RA10: *p* < 0.01; RA30: *p* < 0.001; L‐met + RA10: *p* < 0.01; and L‐met + RA30: *p <* 0.01, paired Student's *t*‐test). Similarly, the PI was significantly higher than 50% chance level in control, RA (10 and 30 mg/kg), and L‐met + RA (10 and 30 mg/kg) groups (Control: *p <* 0.01; RA10: *p <* 0.001; RA30: *p <* 0.0001; L‐met + RA10: *p <* 0.0001; and L‐met + RA30: *p <* 0.01, one‐sample *t*‐test; Figure 3), but not in the L‐met group (*p* > 0.05). These results indicate that L‐met induces recognition memory deficit, which is attenuated by RA treatment.

**FIGURE 3:**
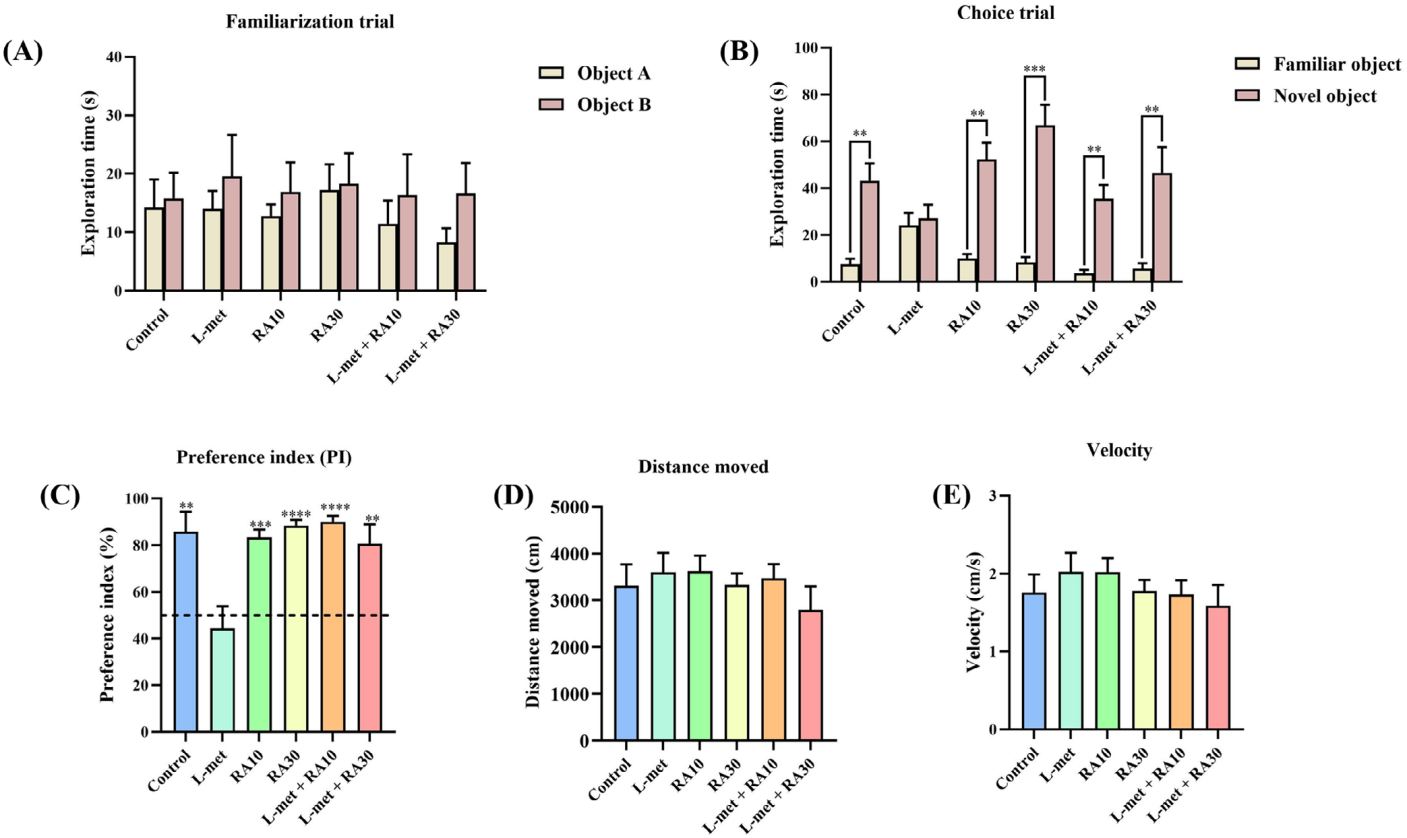
Exploration time during the familiarization (A) and choice trials (B) in the NOR test. No significant differences were observed during the familiarization trial (*p* > 0.05). In the choice trial, all groups except the L‐met group showed a preference for the novel object (**p < 0.01 and ***p < 0.001). The PI (C) in all groups except the L‐met group was significantly higher than the 50% chance level (**p < 0.01, ***p < 0.001, and ****p < 0.0001). Distance moved (D) and velocity (E) revealed no statistically significant differences among groups (*p* > 0.05). Data are presented as mean ± SEM.

### 3.2 Effects of RA and L‐Met on Cell Proliferation and Vascular‐Associated Proliferating Cells

The number of total Ki‐67‐positive cells differed significantly among groups (*F* (5, 30) = 366.8, *p* < 0.0001, one‐way ANOVA; Figure 4, Table 2). The L‐met group revealed a significantly lower number of total Ki‐67‐positive cells compared to the control group (*p* < 0.0001). Moreover, co‐administration of RA (10 and 30 mg/kg) with L‐met significantly increased total Ki‐67‐positive cells compared to the L‐met group (*p* < 0.0001 for both doses).

**FIGURE 4:**
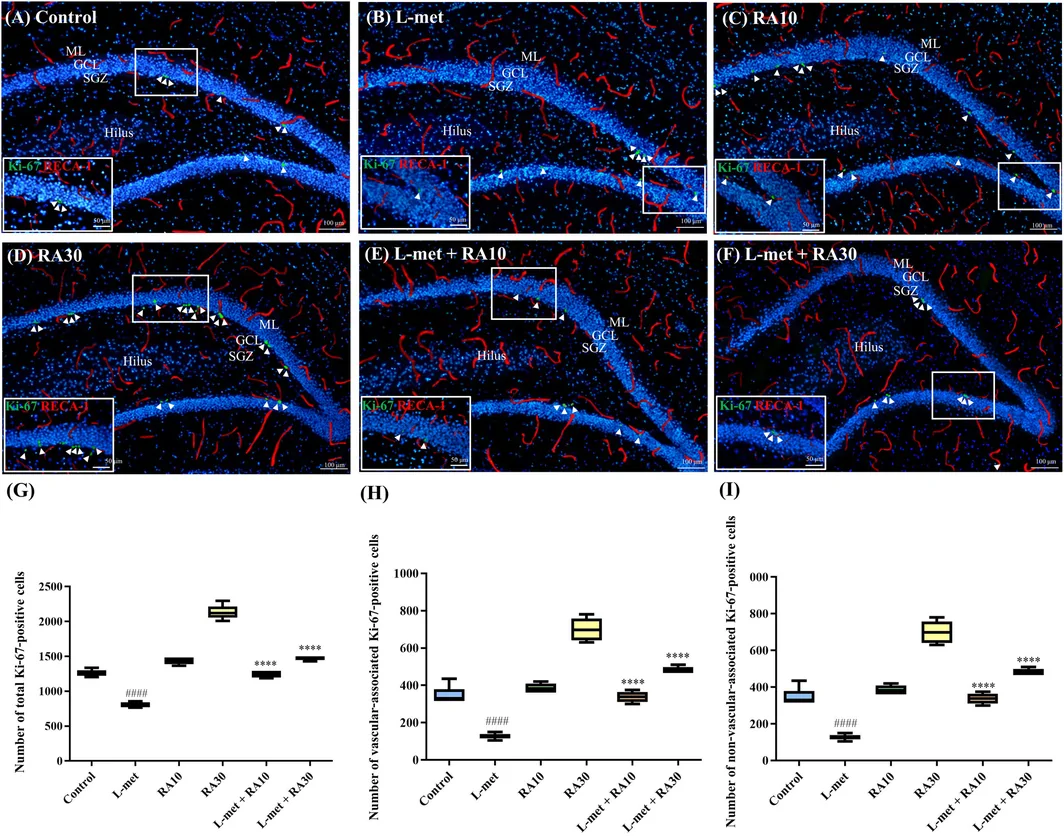
Representative images of Ki‐67‐positive (green) and RECA‐1‐positive (red) cells in the subgranular zone (SGZ) of the dentate gyrus (DG) of the hippocampus across experimental groups (A–F). Nuclei were counterstained with DAPI (blue). Arrowheads indicate Ki‐67‐positive cells. Quantitative analysis of total Ki‐67‐positive cells (G), vascular‐associated Ki‐67‐positive cells (H), and non‐vascular‐associated Ki‐67‐positive cells (I) is shown. Data are presented as mean ± SEM. ^####^ *p* < 0.0001 compared to control group; ****p < 0.0001 compared to L‐met group.

**TABLE 2:** Quantification of Ki‐67‐positive cells in the subgranular zone (SGZ) of the dentate gyrus (DG) of the hippocampus.

Group	Total Ki‐67‐positive cells	Vascular‐associated Ki‐67‐positive cells	Non‐vascular‐associated Ki‐67‐positive cells
Control	1263 ± 19.14	347.5 ± 19.14	915.0 ± 27.11
L‐met	802.5 ± 14.87^####^	127.5 ± 6.42^####^	675.0 ± 11.62^####^
RA10	1433 ± 19.65	385.0 ± 10.00	1048 ± 10.55
RA30	2145 ± 38.73	700.0 ± 25.59	1435 ± 39.62
L‐met + RA10	1240 ± 18.03****	337.5 ± 11.46****	902.5 ± 13.09****
L‐met + RA30	1473 ± 11.88****	482.5 ± 8.14****	990.0 ± 10.25****

*Note:* Data are represented as mean ± SEM. Significance levels compared to control group: ^####^ *p* < 0.0001. Significance levels compared to the L‐met group: ****p < 0.0001. Statistical analysis by 1‐way ANOVA followed by Bonferroni's post hoc test.

The number of vascular‐associated Ki‐67‐positive cells also differed significantly among groups (*F* (5, 30) = 157.0, *p* < 0.0001, one‐way ANOVA; Figure 4, Table 2). The number of vascular‐associated Ki‐67‐positive cells was significantly reduced in the L‐met group compared to the control group (*p* < 0.0001). Furthermore, co‐administration of RA (10 and 30 mg/kg) significantly increased vascular‐associated Ki‐67‐positive cells compared to the L‐met group (*p* < 0.0001 for both doses).

Similarly, the number of non‐vascular‐associated Ki‐67‐positive cells differed significantly among groups (*F* (5, 30) = 133.1, *p* < 0.0001, one‐way ANOVA; Figure 4, Table 2). The L‐met group exhibited a significantly lower number of non‐vascular‐associated Ki‐67‐positive cells compared to the control group (*p* < 0.0001). Co‐administration of RA (10 and 30 mg/kg) significantly increased non‐vascular‐associated Ki‐67‐positive cells compared to the L‐met group (*p* < 0.0001 for both doses).

### 3.3 Effects of RA and L‐Met on Neuronal Cell Survival

The number of BrdU/NeuN‐positive cells differed significantly among groups (*F* (5, 30) = 63.88, one‐way ANOVA, *p* < 0.0001; Figure 5). The L‐met group showed a significant reduction in the number of BrdU/NeuN‐positive cells compared to the control group (*p* < 0.0001), indicating impaired neuronal survival. Furthermore, co‐administration of RA (10 and 30 mg/kg) with L‐met significantly increased the number of BrdU/NeuN‐positive cells compared to the L‐met group (*p* < 0.0001 for both doses), suggesting that RA attenuates L‐met‐induced neuronal loss.

**FIGURE 5:**
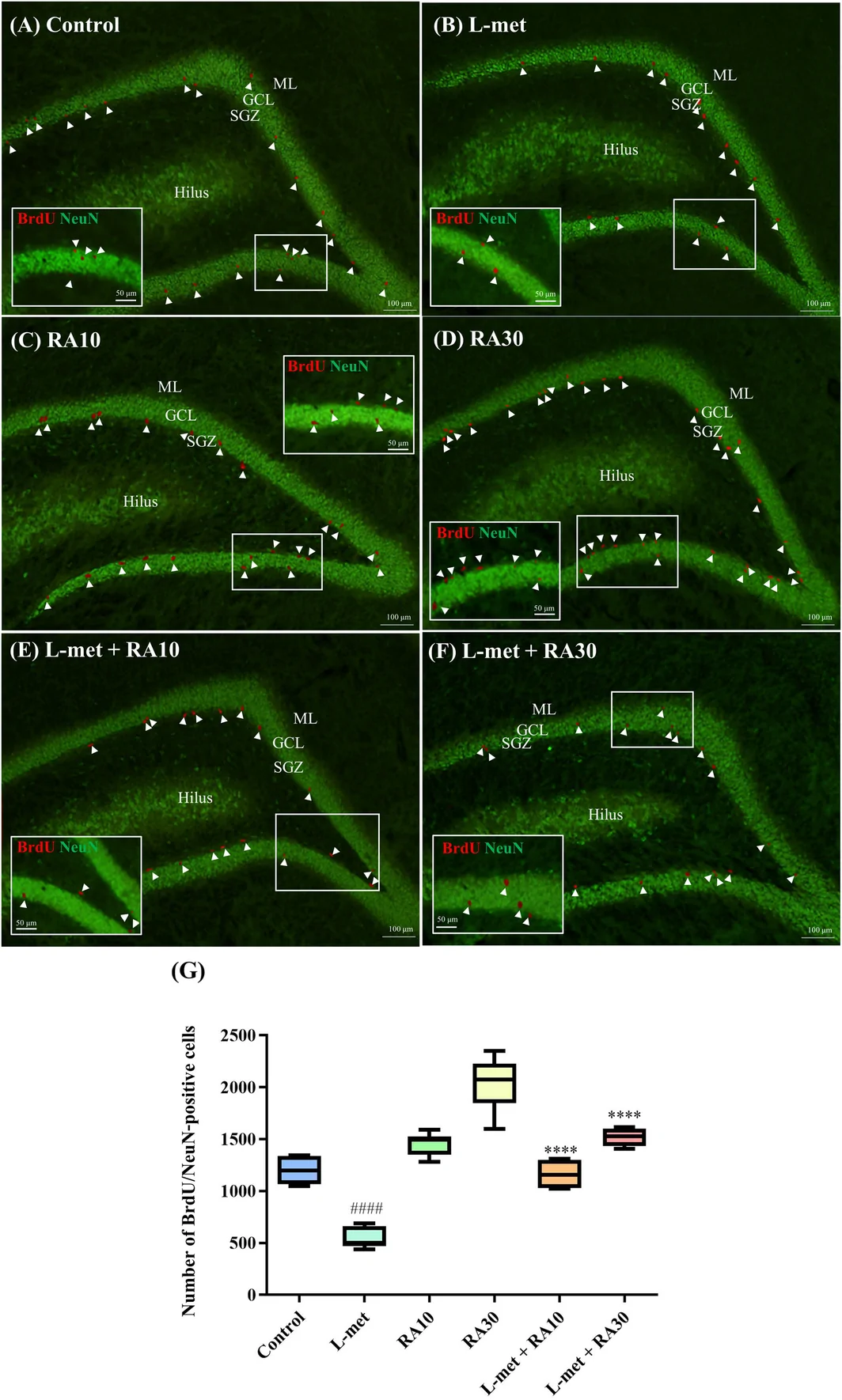
Representative images of BrdU/NeuN double‐labeled cells in the subgranular zone (SGZ) of the hippocampal dentate gyrus (DG) across experimental groups (A–F). BrdU‐positive cells are shown in red and NeuN‐positive cells are shown in green. Arrowheads indicate BrdU/NeuN‐positive cells. Quantitative analysis (G) is presented as mean ± SEM. ^####^ *p* < 0.0001 compared to control group; ****p < 0.0001 compared to L‐met group.

### 3.4 Effects of RA and L‐Met on Cell Cycle Arrest

The number of p21‐positive cells differed significantly among groups (*F* (5, 30) = 43.24, *p* < 0.0001, one‐way ANOVA; Figure 6). The L‐met group showed a significant increase in the number of p21‐positive cells compared to the control group (*p* < 0.0001). Importantly, co‐administration of RA (10 and 30 mg/kg) with L‐met significantly reduced the number of p21‐positive cells compared to the L‐met group (*p* < 0.01 and *p* < 0.0001, respectively).

**FIGURE 6:**
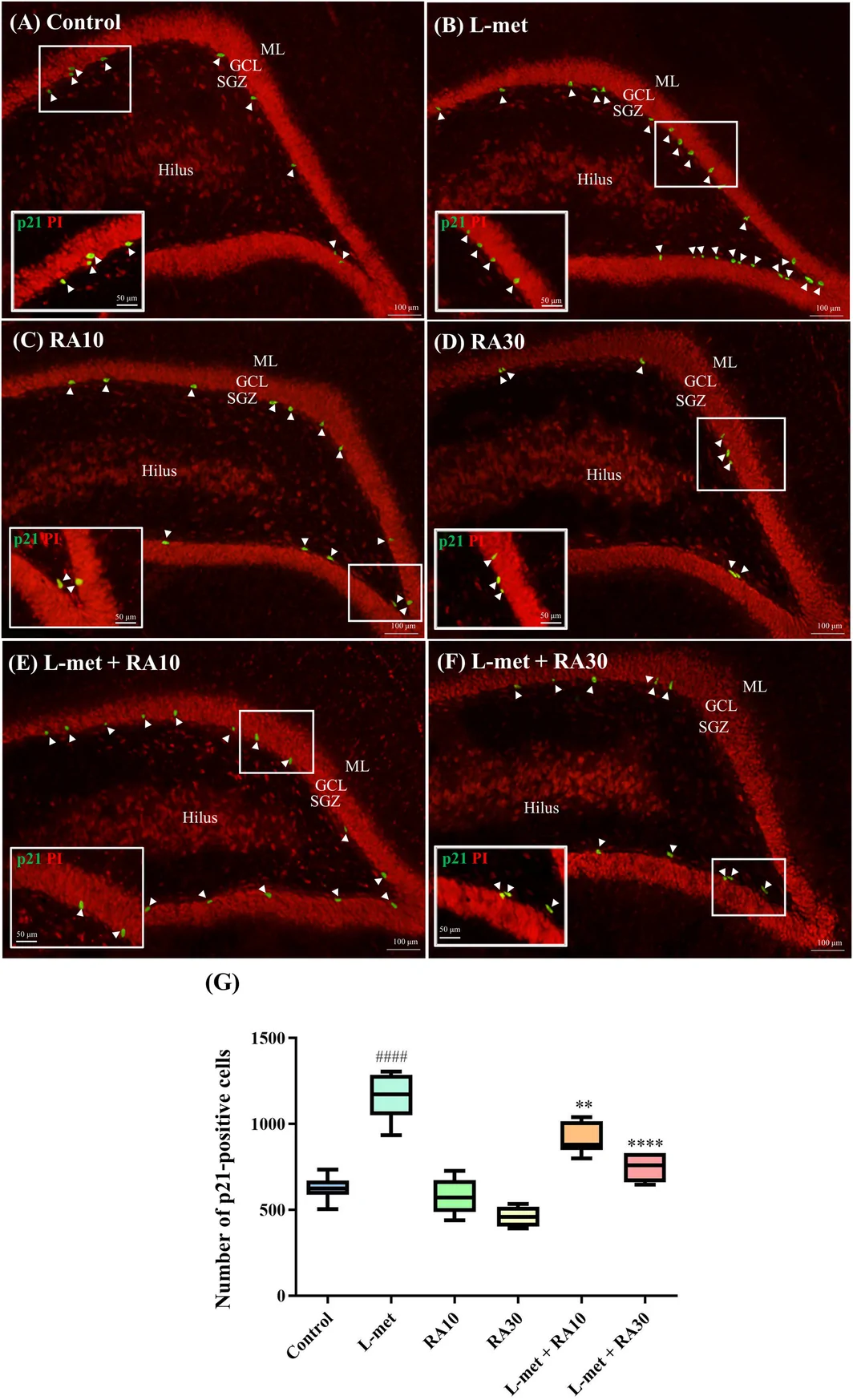
Representative images of p21‐positive cells (green) in the subgranular zone (SGZ) of the dentate gyrus (DG) of the hippocampus across experimental groups (A–F). Nuclei were counterstained with propidium iodide (PI; red). Arrowheads indicate p21‐positive cells. Quantitative analysis (G) is presented as mean ± SEM. ^####^ *p* < 0.0001 compared to control group; **p < 0.01 and ****p < 0.0001 compared to L‐met group.

## 4 Discussion

The present study demonstrated that L‐met administration was associated with impaired hippocampal neurogenesis and cognitive function, as indicated by reduced cell proliferation and survival of newly generated neuronal cells, increased cell‐cycle arrest, and impaired memory function in rats. However, co‐administration of RA attenuated the L‐met‐induced impairments in hippocampal neurogenesis and memory function. In the present study, memory function was assessed using the NOL and NOR tests, while locomotor activity was evaluated based on distance moved and velocity. The results revealed no significant differences in locomotor activity among the groups, indicating that the observed memory deficits were unlikely to be attributable to alterations in general motor function. Rats in the L‐met group exhibited memory deficits in both the NOL and NOR tests, consistent with previous findings of impaired cognitive performance following L‐met treatment (Sirichoat et al. 2024). Conversely, co‐treatment with RA attenuated L‐met‐induced memory deficits, consistent with previous studies reporting that RA may improve learning and memory in experimental models (Ali and Zahid 2020; Fonteles et al. 2016).

Hippocampal neurogenesis is closely associated with memory formation and spatial navigation within a vascularized neurogenic microenvironment (Djoher Nora Abrous and Wojtowicz 2015; Shen et al. 2004). Our results showed that L‐met significantly reduced the number of Ki‐67‐ and Ki‐67/RECA‐1‐positive cells in the DG, consistent with previous findings that L‐met negatively affects neural cell proliferation and vascular‐associated neurogenesis (Dornlakorn et al. 2026; Sirichoat et al. 2024). Previous studies have reported that chronic L‐met administration can induce HHcy in rodents, while elevated homocysteine levels have been associated with cognitive impairment and neurotoxicity (Dornlakorn et al. 2026; Obeid and Herrmann 2006; Sudduth et al. 2013). In the present study, co‐administration of RA with L‐met attenuated the reduction in Ki‐67‐ and Ki‐67/RECA‐1‐positive cells, suggesting that RA may help preserve hippocampal cell proliferation and vascular‐associated proliferating cells. This is consistent with evidence that RA promotes cell proliferation and neurogenesis (Ali and Zahid 2020).

We also investigated the survival of newly generated neuronal cells using BrdU/NeuN staining. The present study showed that L‐met treatment decreased the number of BrdU/NeuN‐positive cells in the DG, suggesting a reduction in the survival of newly generated neuronal cells. Previous studies have reported that elevated homocysteine levels are associated with decreased brain‐derived neurotrophic factor (BDNF) expression, which may affect neurotrophic signaling and neuronal survival (Dornlakorn et al. 2026; Wei et al. 2023). In contrast, co‐treatment with RA attenuated the reduction in BrdU/NeuN‐positive cells induced by L‐met, suggesting that RA may attenuate the L‐met‐induced reduction in the survival of newly generated neuronal cells. Previous studies have reported that RA may promote neurogenesis through BDNF‐related signaling (Zeng et al. 2024).

L‐met administration has been associated with oxidative stress and cellular stress, including inflammation, DNA damage, and cell death, which may contribute to cognitive impairment (Dornlakorn et al. 2026; Obeid and Herrmann 2006; Sudduth et al. 2013). Our study demonstrated that L‐met treatment significantly increased p21‐positive cells in the DG, suggesting increased cell cycle arrest in the DG. Increased p21 expression has been associated with inhibition of neural progenitor cell‐cycle progression, thereby reducing cell proliferation and impairing hippocampal neurogenesis (Kaewngam et al. 2024; Prajit et al. 2024; Umka Welbat et al. 2016). This is consistent with our findings that increased p21‐positive cells were accompanied by reduced Ki‐67‐ and BrdU/NeuN‐positive cells in the DG. In contrast, co‐treatment with RA significantly attenuated L‐met‐induced increase in p21‐positive cells. The reduction in p21‐positive cells following RA co‐administration was associated with the preservation of hippocampal cell proliferation and survival. These findings suggest that the effects of RA on hippocampal neurogenesis may be associated with reduced cell‐cycle arrest. Previous studies have reported that RA may attenuate oxidative stress and neuroinflammatory responses in experimental models (Hassanzadeh‐Taheri et al. 2021; Luan et al. 2013; Wang, Wang, et al. 2021).

In conclusion, L‐met induced cognitive impairment, as demonstrated by behavioral testing, and was associated with reduced hippocampal neurogenesis and increased p21‐positive cells, suggesting enhanced cell‐cycle arrest. However, co‐administration of RA attenuated these impairments, as evidenced by preservation of hippocampal cell proliferation and survival of newly generated neuronal cells and the reduction in p21‐positive cells in the DG. These findings suggest that RA may attenuate L‐met‐induced cognitive impairment.

### 4.1 Limitations

Homocysteine, oxidative stress, apoptotic, and BDNF/TrkB‐related signaling were not evaluated, and the mechanisms underlying p21‐associated cell‐cycle arrest were not directly investigated. Therefore, the involvement of these pathways in the effects of L‐met and RA cannot be confirmed based on the present findings. Future studies should investigate these biochemical and molecular pathways to better understand the mechanisms underlying the effects of L‐met and RA.

## Author Contributions

**Salinee Naewla:** conceptualization, methodology, data curation, writing – review and editing, investigation, formal analysis. **Jirawadee Chaithum:** investigation, methodology, formal analysis, data curation. **Tanaporn Anosri:** investigation, formal analysis. **Nataya Sritawan:** investigation, formal analysis. **Anusara Aranarochana:** investigation, formal analysis. **Jariya Umka Welbat:** supervision, methodology, validation, project administration. **Peter Wigmore:** writing – original draft, project administration. **Apiwat Sirichoat:** project administration, conceptualization, methodology, validation, resources, funding acquisition, writing – review and editing.

## Funding

This work was supported by The National Science, Research, and Innovation Fund through the Fundamental Fund of Khon Kaen University, NSRF‐KKU: 200595, 2024. The Postgraduate Study Support Grant of the Faculty of Medicine, Khon Kaen University, Khon Kaen, Thailand.

## Ethics Statement

This study was approved by the Animal Ethics Committee of Khon Kaen University (AICUC‐KKU‐70/2565).

## Conflicts of Interest

The authors declare no conflicts of interest.

## Data Availability

The data that support the findings of this study are available on request from the corresponding author. The data are not publicly available due to privacy or ethical restrictions.

## References

[fsn372502-bib-0001] Abrous, D. N. , M. Koehl , and M. Le Moal . 2005. “Adult Neurogenesis: From Precursors to Network and Physiology.” Physiological Reviews 85: 523–569. 10.1152/physrev.00055.2003. 15788705

[fsn372502-bib-0002] Abrous, D. N. , and J. M. Wojtowicz . 2015. “Interaction Between Neurogenesis and Hippocampal Memory System: New Vistas.” Cold Spring Harbor Perspectives in Biology 7: a018952. 10.1101/cshperspect.a018952. PMC4448601 26032718

[fsn372502-bib-0003] Adomako‐Bonsu, A. G. , S. L. Chan , M. Pratten , and J. R. Fry . 2017. “Antioxidant Activity of Rosmarinic Acid and Its Principal Metabolites in Chemical and Cellular Systems: Importance of Physico‐Chemical Characteristics.” Toxicology In Vitro 40: 248–255. 10.1016/j.tiv.2017.01.016. 28122265

[fsn372502-bib-0004] Ali, M. , and S. Zahid . 2020. “The Neurogenic Effects of Rosmarinic Acid in a Mouse Model of Type 2 Diabetes Mellitus.” Brazilian Journal of Pharmaceutical Sciences 56: e18772.

[fsn372502-bib-0005] Amoah, S. K. , L. P. Sandjo , J. M. Kratz , and M. W. Biavatti . 2016. “Rosmarinic Acid—Pharmaceutical and Clinical Aspects.” Planta Medica 82: 388–406. 10.1055/s-0035-1568274. 26845712

[fsn372502-bib-0006] Bottiglieri, T. 2002. “S‐Adenosyl‐L‐Methionine (SAMe): From the Bench to the Bedside—Molecular Basis of a Pleiotrophic Molecule.” American Journal of Clinical Nutrition 76: 1151–1157. 10.1093/ajcn/76/5.1151S. 12418493

[fsn372502-bib-0007] Cam, J. A. , and G. Bu . 2006. “Modulation of Beta‐Amyloid Precursor Protein Trafficking and Processing by the Low Density Lipoprotein Receptor Family.” Molecular Neurodegeneration 1: 8. 10.1186/1750-1326-1-8. PMC1563464 16930455

[fsn372502-bib-0008] Dix, S. L. , and J. P. Aggleton . 1999. “Extending the Spontaneous Preference Test of Recognition: Evidence of Object‐Location and Object‐Context Recognition.” Behavioural Brain Research 99: 191–200. 10.1016/s0166-4328(98)00079-5 10512585

[fsn372502-bib-0009] Dornlakorn, O. , A. Sirichoat , W. Pannangrong , et al. 2026. “Caffeic Acid Ameliorates L‐Methionine‐Induced Hyperhomocysteinemia, Leading to Decreased Adult Neurogenesis and Oxidative Stress Status in Adult Rats.” ACS Chemical Neuroscience 17: 2379–2388. 10.1021/acschemneuro.6c00206 PMC13281375 42216877

[fsn372502-bib-0010] Eriksson, P. S. , E. Perfilieva , T. Björk‐Eriksson , et al. 1998. “Neurogenesis in the Adult Human Hippocampus.” Nature Medicine 4: 1313–1317. 10.1038/3305. 9809557

[fsn372502-bib-0011] Fonteles, A. A. , C. M. de Souza , J. C. de Sousa Neves , et al. 2016. “Rosmarinic Acid Prevents Against Memory Deficits in Ischemic Mice.” Behavioural Brain Research 297: 91–103. 10.1016/j.bbr.2015.09.029. 26456521

[fsn372502-bib-0012] Hasanein, P. , R. Seifi , M. R. Hajinezhad , and A. Emamjomeh . 2017. “Rosmarinic Acid Protects Against Chronic Ethanol‐Induced Learning and Memory Deficits in Rats.” Nutritional Neuroscience 20: 547–554. 10.1080/1028415x.2016.1203125. 27367870

[fsn372502-bib-0013] Hase, T. , S. Shishido , S. Yamamoto , et al. 2019. “Rosmarinic Acid Suppresses Alzheimer's Disease Development by Reducing Amyloid β Aggregation by Increasing Monoamine Secretion.” Scientific Reports 9: 8711. 10.1038/s41598-019-45168-1. PMC6581955 31213631

[fsn372502-bib-0014] Hassanzadeh‐Taheri, M. , A. Ahmadi‐Zohan , M. Mohammadifard , and M. Hosseini . 2021. “Rosmarinic Acid Attenuates Lipopolysaccharide‐Induced Neuroinflammation and Cognitive Impairment in Rats.” Journal of Chemical Neuroanatomy 117: 102008. 10.1016/j.jchemneu.2021.102008. 34314849

[fsn372502-bib-0015] Hemanth Kumar, B. , B. Dinesh Kumar , and P. V. Diwan . 2017. “Hesperidin, a Citrus Flavonoid, Protects Against l‐Methionine‐Induced Hyperhomocysteinemia by Abrogation of Oxidative Stress, Endothelial Dysfunction and Neurotoxicity in Wistar Rats.” Pharmaceutical Biology 55: 146–155. 10.1080/13880209.2016.1231695. PMC7011910 27677544

[fsn372502-bib-0016] Hernández‐Mercado, K. , and A. Zepeda . 2021. “Morris Water Maze and Contextual Fear Conditioning Tasks to Evaluate Cognitive Functions Associated With Adult Hippocampal Neurogenesis.” Frontiers in Neuroscience 15: 782947. 10.3389/fnins.2021.782947. PMC8761726 35046769

[fsn372502-bib-0017] Ingenbleek, Y. , and H. Kimura . 2013. “Nutritional Essentiality of Sulfur in Health and Disease.” Nutrition Reviews 71: 413–432. 10.1111/nure.12050. 23815141

[fsn372502-bib-0018] Kaewngam, S. , R. Prajit , T. Anosri , et al. 2024. “The Effects of Hesperidin on Valproic Acid‐Induced Reduction in Hippocampal Neurogenesis Through the Antioxidant and Apoptotic Pathways in Adult Rats.” Scientific Reports 14: 28864. 10.1038/s41598-024-80183-x. PMC11582586 39572680

[fsn372502-bib-0019] Kalhan, S. C. , and S. E. Marczewski . 2012. “Methionine, Homocysteine, One Carbon Metabolism and Fetal Growth.” Reviews in Endocrine and Metabolic Disorders 13: 109–119. 10.1007/s11154-012-9215-7. 22418620

[fsn372502-bib-0020] Kohman, R. A. , and J. S. Rhodes . 2013. “Neurogenesis, Inflammation and Behavior.” Brain, Behavior, and Immunity 27: 22–32. 10.1016/j.bbi.2012.09.003. PMC3518576 22985767

[fsn372502-bib-0021] Kovalska, M. , E. Baranovicova , D. Kalenska , et al. 2021. “Methionine Diet Evoked Hyperhomocysteinemia Causes Hippocampal Alterations, Metabolomics Plasma Changes and Behavioral Pattern in Wild Type Rats.” International Journal of Molecular Sciences 22: 4961. 10.3390/ijms22094961. PMC8124831 34066973

[fsn372502-bib-0022] Lee, J. , W. Duan , and M. P. Mattson . 2002. “Evidence That Brain‐Derived Neurotrophic Factor Is Required for Basal Neurogenesis and Mediates, in Part, the Enhancement of Neurogenesis by Dietary Restriction in the Hippocampus of Adult Mice.” Journal of Neurochemistry 82: 1367–1375. 10.1046/j.1471-4159.2002.01085.x. 12354284

[fsn372502-bib-0023] Luan, H. , Z. Kan , Y. Xu , C. Lv , and W. Jiang . 2013. “Rosmarinic Acid Protects Against Experimental Diabetes With Cerebral Ischemia: Relation to Inflammation Response.” Journal of Neuroinflammation 10: 28. 10.1186/1742-2094-10-28. PMC3614882 23414442

[fsn372502-bib-0024] Millán‐Calenti, J. C. , J. Tubío , S. Pita‐Fernández , I. González‐Abraldes , T. Lorenzo , and A. Maseda . 2009. “Prevalence of Cognitive Impairment: Effects of Level of Education, Age, Sex and Associated Factors.” Dementia and Geriatric Cognitive Disorders 28: 455–460. 10.1159/000257086. 19907183

[fsn372502-bib-0025] Naewla, S. , A. Sirichoat , W. Pannangrong , P. Chaisawang , P. Wigmore , and J. U. Welbat . 2019. “Hesperidin Alleviates Methotrexate‐Induced Memory Deficits via Hippocampal Neurogenesis in Adult Rats.” Nutrients 11: 936. 10.3390/nu11040936. PMC6521088 31027240

[fsn372502-bib-0026] Obeid, R. , and W. Herrmann . 2006. “Mechanisms of Homocysteine Neurotoxicity in Neurodegenerative Diseases With Special Reference to Dementia.” FEBS Letters 580: 2994–3005. 10.1016/j.febslet.2006.04.088. 16697371

[fsn372502-bib-0027] Orgeron, M. L. , K. P. Stone , D. Wanders , C. C. Cortez , N. T. Van , and T. W. Gettys . 2014. “The Impact of Dietary Methionine Restriction on Biomarkers of Metabolic Health.” Progress in Molecular Biology and Translational Science 121: 351–376. 10.1016/B978-0-12-800101-1.00011-9 PMC4049285 24373243

[fsn372502-bib-0028] Park, M. H. , D. Y. Kwon , J. M. Jung , C. Han , I. Jo , and S. A. Jo . 2013. “Mini‐Mental Status Examination as Predictors of Mortality in the Elderly.” Acta Psychiatrica Scandinavica 127: 298–304. 10.1111/j.1600-0447.2012.01918.x. 22901036

[fsn372502-bib-0029] Parle, M. , and N. Singh . 2007. “Reversal of Memory Deficits by Atorvastatin and Simvastatin in Rats.” Yakugaku Zasshi 127: 1125–1137. 10.1248/yakushi.127.1125. 17603272

[fsn372502-bib-0030] Prajit, R. , R. Saenno , K. Suwannakot , et al. 2024. “Chrysin Mitigates Neuronal Apoptosis and Impaired Hippocampal Neurogenesis in Male Rats Subjected to D‐Galactose‐Induced Brain Aging.” Biogerontology 25: 1275–1284. 10.1007/s10522-024-10140-8. PMC11486779 39300009

[fsn372502-bib-0031] Shen, Q. , S. K. Goderie , L. Jin , et al. 2004. “Endothelial Cells Stimulate Self‐Renewal and Expand Neurogenesis of Neural Stem Cells.” Science 304: 1338–1340. 10.1126/science.1095505. 15060285

[fsn372502-bib-0032] Shin, J. H. 2022. “Dementia Epidemiology Fact Sheet 2022.” Annals of Rehabilitation Medicine 46: 53–59. 10.5535/arm.22027. PMC9081392 35508924

[fsn372502-bib-0033] Sirichoat, A. , O. Dornlakorn , R. Saenno , et al. 2024. “Caffeic Acid Protects Against L‐Methionine Induced Reduction in Neurogenesis and Cognitive Impairment in a Rat Model.” Heliyon 10: e26919. 10.1016/j.heliyon.2024.e26919. PMC10918208 38455532

[fsn372502-bib-0034] Sudduth, T. L. , D. K. Powell , C. D. Smith , A. Greenstein , and D. M. Wilcock . 2013. “Induction of Hyperhomocysteinemia Models Vascular Dementia by Induction of Cerebral Microhemorrhages and Neuroinflammation.” Journal of Cerebral Blood Flow & Metabolism 33: 708–715. 10.1038/jcbfm.2013.1. PMC3652696 23361394

[fsn372502-bib-0035] Umka, J. , S. Mustafa , M. ElBeltagy , et al. 2010. “Valproic Acid Reduces Spatial Working Memory and Cell Proliferation in the Hippocampus.” Neuroscience 166: 15–22. 10.1016/j.neuroscience.2009.11.073. 20006675

[fsn372502-bib-0036] Umka Welbat, J. , A. Sirichoat , W. Chaijaroonkhanarak , et al. 2016. “Asiatic Acid Prevents the Deleterious Effects of Valproic Acid on Cognition and Hippocampal Cell Proliferation and Survival.” Nutrients 8: 303. 10.3390/nu8050303. PMC4882715 27213437

[fsn372502-bib-0037] Wang, C. C. , P. W. Hsieh , J. R. Kuo , and S. J. Wang . 2021. “Rosmarinic Acid, a Bioactive Phenolic Compound, Inhibits Glutamate Release From Rat Cerebrocortical Synaptosomes Through GABA(A) Receptor Activation.” Biomolecules 11: 1029. 10.3390/biom11071029. PMC8301814 34356653

[fsn372502-bib-0038] Wang, J. , S. Wang , H. Guo , et al. 2021. “Rosmarinic Acid Protects Rats Against Post‐Stroke Depression After Transient Focal Cerebral Ischemic Injury Through Enhancing Antioxidant Response.” Brain Research 1757: 147336. 10.1016/j.brainres.2021.147336. 33548269

[fsn372502-bib-0039] Wang, J. , H. Xu , H. Jiang , X. Du , P. Sun , and J. Xie . 2012. “Neurorescue Effect of Rosmarinic Acid on 6‐Hydroxydopamine‐Lesioned Nigral Dopamine Neurons in Rat Model of Parkinson's Disease.” Journal of Molecular Neuroscience 47: 113–119. 10.1007/s12031-011-9693-1. 22205146

[fsn372502-bib-0040] Wei, M. D. , Y. Y. Huang , Y. Zeng , et al. 2023. “Homocysteine Modulates Social Isolation‐Induced Depressive‐Like Behaviors Through BDNF in Aged Mice.” Molecular Neurobiology 60: 4924–4934. 10.1007/s12035-023-03377-w. PMC10191402 37198386

[fsn372502-bib-0041] Yamamoto, S. , T. Kayama , M. Noguchi‐Shinohara , T. Hamaguchi , and M. Yamada . 2021. “Rosmarinic Acid Suppresses Tau Phosphorylation and Cognitive Decline by Downregulating the JNK Signaling Pathway.” npj Science of Food 5: 1. 10.1038/s41538-021-00084-5. PMC7846760 33514742

[fsn372502-bib-0042] Zeng, J. , Z. Xie , L. Chen , et al. 2024. “Rosmarinic Acid Alleviate CORT‐Induced Depressive‐Like Behavior by Promoting Neurogenesis and Regulating BDNF/TrkB/PI3K Signaling Axis.” Biomedicine & Pharmacotherapy 170: 115994. 10.1016/j.biopha.2023.115994. 38070249

